# Cannabinoid 1 receptor availability in posttraumatic stress disorder: A positron emission tomography study

**DOI:** 10.1038/s41398-025-03519-9

**Published:** 2025-08-22

**Authors:** Nachshon Korem, Anahita Bassir Nia, Ansel T. Hillmer, Deepak D’Souza, Nabeel Nabulsi, Jim Ropchan, Yiyun Huang, Kelly Cosgrove, Ifat Levy, Robert H. Pietrzak, Ilan Harpaz-Rotem

**Affiliations:** 1 Department of Psychiatry, Yale University School of Medicine, New Haven, CT USA; 2 U.S. Department of Veterans Affairs National Center for Posttraumatic Stress Disorder, VA Connecticut Healthcare System, West Haven, CT USA; 3 Department of Comparative Medicine, Yale University School of Medicine, New Haven, CT USA; 4 Radiology and Biomedical Imaging, Yale School of Medicine, New Haven, CT USA; 5 Department of Biomedical Engineering, Yale School of Engineering and Applied Sciences, Yale University, New Haven, CT USA; 6 PET Center, Department of Radiology and Biomedical Imaging, Yale University School of Medicine, New Haven, CT USA; 7 Wu Tsai Institute, Yale University, New Haven, CT USA; 8 Department of Psychology, Yale University, New Haven, CT USA; 9 Department of Neuroscience, Yale University, New Haven, CT USA; 10 Yale School of Public Health, New Haven, CT USA

**Keywords:** Neuroscience, Psychiatric disorders

## Abstract

The endocannabinoid system (ECS) plays a critical role in fear learning and maintenance and may, therefore, be implicated in the pathophysiology of posttraumatic stress disorder (PTSD). The exact role of cannabinoid receptor 1 (CB1R), a key component of the ECS, remains unclear. Although preclinical studies largely suggest CB1R downregulation in PTSD, the only prior study of CB1R availability in individuals with PTSD reported higher levels than in controls. In this study, we investigated the relationship between CB1R availability and PTSD diagnosis and symptoms. Using positron emission tomography (PET) with the CB1R-specific radiotracer [^11^C]OMAR, scans from 62 individuals, including 46 trauma-exposed participants (19 with current PTSD) and 16 healthy controls, were analyzed. Our findings revealed no differences in CB1R availability between groups in either the whole brain or regions of interest. However, emotional numbing symptoms of PTSD were significantly linked to CB1R availability. These results suggest that the ECS role in the maintenance of PTSD is more nuanced than previously suggested. The ECS was linked to specific PTSD symptom expression, highlighting the potential for treatments targeting the ECS in mitigating these specific symptoms of this multi-faceted disorder.

## Introduction

Posttraumatic stress disorder (PTSD) is a debilitating psychiatric condition that arises following exposure to traumatic events [1]. Converging evidence suggests that the endocannabinoid system (ECS) plays a crucial role in the pathophysiology of PTSD [2]. The ECS, through its receptors, cannabinoid receptors 1 (CB1R) and 2 (CB2R), and its endogenous ligands, anandamide (AEA) and 2-arachidonoylglycerol (2-AG), regulate processes involved in stress response, emotional regulation, and memory consolidation [3]. Most importantly, CB1R are highly expressed in brain regions implicated in fear and stress responses (e.g., the amygdala, hippocampus, and prefrontal cortex) and, thus, are particularly interesting in the study of the development, maintenance [4, 5], and the treatment of fear-related disorders such as PTSD [2, 6, 7].

The ECS plays a key inhibitory role in the central nervous system by regulating neurotransmission in a retrograde manner [8]. Specifically, endocannabinoids are released from the postsynaptic neuron to inhibit the presynaptic neuron by blocking calcium channels, reducing neurotransmitter release. In response to acute threats, AEA levels decrease, facilitating the recruitment of glucocorticoids [9, 10]. This is followed by a sharp increase in 2-AG levels, which helps restore homeostasis by regulating the stress response. The ECS is highly dynamic, with receptor and ligand levels adapting to meet physiological demands [11]. Nevertheless, chronic increases in endocannabinoids or cannabinoids, such as in models of chronic stress or people with cannabis use disorder, lead to a reduction in CB1R availability [12–14], probably as an adaptive response to prolonged activation.

In PTSD, a condition characterized by a persistent sense of threat [15], the only study investigating CB1R availability reported higher CB1R availability compared to healthy controls, along with reduced AEA levels [16]. This finding is consistent with some preclinical rodent studies, which observed elevated CB1R expression in key brain regions following intense foot shocks [17, 18]. However, animal models also reported contradictory evidence, with some studies reporting reduced CB1R mRNA expression in key regions in PTSD models, such as predator stress paradigms [13, 19, 20]. Furthermore, animal models of chronic unpredictable stress (CUS) reliably demonstrated that multiple exposure to traumatic events results in lower levels of CB1R in stress-related brain regions such as the hippocampus[21, 22]. Similarly, a reduction in CB1R in the hippocampus is reported in animal models of childhood trauma, such as maternal deprivation [23, 24]. Moreover, studies on the peripheral levels of endocannabinoids, with clinical and preclinical populations, produced contradictory results, reporting lower [16, 25, 26], higher [27], or observing no difference between individuals with PTSD and healthy controls [7]. Of note, the investigation of ECS in other psychiatric disorders indicated lower CB1R [28, 29], adding to the uncertainty surrounding the role of ECS in PTSD.

Given these conflicting findings, and with only a single study on CB1R availability in humans exposed to trauma, the current study aimed to investigate the relationship between CB1R availability and PTSD, and to shed further light on the single study done thus far [16]. Here, we aim to compare CB1R availability between individuals diagnosed with PTSD, trauma-exposed controls (TC), and healthy control individuals (HC). Additionally, we investigated associations between CB1R availability and PTSD symptom severity using the original DSM-5 clusters and the more nuanced 8-cluster model of PTSD [30] to clarify the role of CB1R in PTSD symptomatology.

## Methods

### Participants

Data from 62 individuals were analyzed (see Table 1). Forty-six participants who met criteria A for the diagnosis of PTSD underwent clinical screening using the Structured Clinical Interview for DSM-5 (SCID-5) [31] and the Clinician-Administered PTSD Scale for DSM-5 (CAPS-5) [32]. Based on the latter, *n* = 19 participants met the criteria for chronic PTSD diagnosis (“PTSD” group; more than 1 year since trauma), and *n* = 27 were exposed to trauma but did not meet PTSD diagnosis (“TC” group). In addition, 16 participants who did not meet criteria A and had no Axis I diagnosis were classified as healthy controls (“HC” group). Exclusion criteria included non-affective (major depressive disorder; MDD) major psychiatric or neurological illness, moderate/severe substance use, unstable psychotropic medication, significant bloodwork abnormalities or positive drug screen (participants were required to abstain from cannabis use for at least 28 days prior to the study), contraindications to MRI, pregnancy/lactation, and recent use of unapproved medications, for detailed exclusion criteria, please refer to the Supplementary Methods.

**Table 1:** Demographic and clinical characteristics of study groups.

	*HCs*	*TCs*	*PTSD*		
	*16*	*27*	*19*		
*N*	*M (s.d.) or n (%)*	*M (s.d.) or n (%)*	*M (s.d.) or n (%)*	*Test of difference*	*Pairwise comparisons*
Age	29.37 (8.16)	45.78 (13.90)	42.58 (13.03)	F(2,59) = 9.14, P = 0.001	HC < TC, PTSD
Male sex	9 (56.25%)	25 (92.6%)	10 (52.63%)	*χ*^*2*^(2) = 10.91, P = 0.004	TC > HC, PTSD
Veteran (yes)	0 (0%)	24	11	*χ*^*2*^(2) = 32.31, P = 0.001	
White race/ethnicity	10 (62.5%)	22 (81.5%)	16 (84.2%)	*χ*^*2*^(2) = 3.66, P = 0.16 NS	
Body mass index	25.76 (4.88)	29.74 (6.04)	30.133 (5.79)	F(2,59) = 3.17, P = 0.049	HC < TC, PTSD
*Indices of criteria: A lifetime trauma*					
Age at first trauma	N/A	*n* = 21 16.52 (7.1)	*n* = 10 14.3 (7.96)	T(16) = 0.75,p = 0.46 NS	
Age at presenting trauma	N/A	*n* = 24 29.4 (9.37)	*n* = 18 22.65 (14.6)	T(25) = 1.59, p = 0.12 NS	
Number of traumas	0	*n* = 24 5.29 (3.3)	*n* = 11 27.64 (32.5)	T(10) = 2.27, p < 0.045	
*Index traumatic event (n)*		24	18		
Military trauma	0	20	9		
Physical assault	0	1	9		
Motor vehicle accident	0	2	0		
Witnessed suicide	0	1	0		
Total number of current psychiatric diagnoses (not including PTSD)	0	*n* = 24 0.42 (0.58)	*N* = 19 2 (1.41)		
CAPS-5 score	—	8.72 (6.91)	38.42 (9.34)	T(29) = 11.26, p < 0.001	PTSD > TC
Injected mass	13.71 (3.85)	14.22 (3.34)	12.05 (4.83)	F(2,59) = 1.85, P = 0.17 NS	

### Ethical approval

The study was approved by both Yale University (HIC#2000025067) and the VA Connecticut Healthcare System (HSS#IHR009) Institutional Review Boards. All participants gave informed consent and received monetary compensation for their participation. All the methods and procedures were performed in accordance with the relevant guidelines and regulations.

### Magnetic resonance imaging (MRI) acquisition

MRI data were obtained using a 3 T Siemens Prisma scanner at the Yale Magnetic Resonance Research Center (MRRC), equipped with a 32-channel receiver array head coil. High-resolution structural images were acquired via Magnetization-Prepared Rapid Gradient-Echo (MPRAGE) imaging (TR = 2.5 s, TE = 2.83 ms, FOV = 256 × 256 mm^2^, matrix = 256 × 256 mm^2^, slice thickness = 1.0 mm without gap, 160 slices, voxel size 1.0 × 1.0 × 1.0 mm^3^).

### Radiochemistry and PET image acquisition

[^11^C]OMAR, a radiotracer with high binding affinity and selectivity for brain CB1 receptors [33, 34], was synthesized using established protocols adapted for automated production on the GE TRACERlab FXC-Pro synthesis module (GE Healthcare, Milwaukee, WI, USA) [33]. Mean (SD) molar activity at the time of injection was 179 (113) MBq/nmol, and injected activity was 485 (147) MBq. Participants underwent dynamic PET scans using an HRRT scanner (Siemens Medical Systems, Knoxville, TN). Prior to each PET scan, a transmission scan was acquired with a ^137^Cs point source for attenuation correction. PET emission data acquisition began with the administration of [^11^C]OMAR via bolus injection (i.v.) over 1 min and continued for 120 min. Participant motion was captured with an optical system (Polaris Vicra, Northern Digital Incorporated, Waterloo, Ontario, Canada) positioned behind the PET scanner to record the position and orientation of an infrared reflective tool mounted to the subject’s head.

Radioactivity concentration in arterial whole blood was measured during the initial 7 min following [^11^C]OMAR administration using either continuous measurement with an integrated peristaltic pump and radioactivity detection system (PBS101, Veenstra Instruments, Joure, The Netherlands) or rapid manual sampling. Discrete samples were manually drawn for all scans at specific time points (3, 5, 7, 10, 15, 20, 30, 45, 60, 75, 90, 105, and 120 min post-injection). Gamma counter measurements (Wizard 1480, PerkinElmer, Waltham, MA, USA) determined each sample’s whole blood and plasma radioactivity. This was then converted to concentration based on the sample weight and density. Additionally, samples collected at specific time points (5, 15, 30, 60, 90, and 120 min) were analyzed using column-switching high-performance liquid chromatography (HPLC) [35] to determine the fraction of unmetabolized radiotracer, as previously described [34]. The unmetabolized parent fraction was calculated as the ratio of radioactivity in fractions containing the parent compound to the total radioactivity collected, fitted with an inverted gamma function, and normalized by the time-varying extraction efficiency of radioactivity for the corresponding filtered plasma sample. Finally, the metabolite-corrected arterial plasma input function was obtained by multiplying the total plasma radioactivity concentration curve by the parent fraction curve on a point-by-point basis.

### PET image processing

Dynamic list mode data were reconstructed with corrections for subject motion, attenuation, normalization, scatter, randoms, and dead time using an ordered subset-expectation maximization (OSEM) [36] algorithm (2 iterations, 30 subsets) histogrammed into 33 frames. Post-reconstruction software motion correction was performed on the dynamic images using a mutual-information algorithm (FSL-FLIRT version 3.2, Analysis Group, FMRIB, Oxford, UK) [37], employing frame-by-frame registration to an early summed image (0–10 min post-injection), which was also registered to the subject’s MR anatomical image (6-parameter affine registration). The subject-specific MR image was subsequently registered to the Anatomical Automatic Labeling (AAL) [38] atlas using a non-linear registration routine for the region of interest (ROI) delineation [39]. The final outcome measure was regional [^11^C]OMAR volume of distribution (*V*_T_), referred to as CB1R availability, because it is proportional to the number of CB1R available for [^11^C]OMAR binding [40]. [^11^C]OMAR *V*_T_ was estimated using the multilinear analysis-1 method (MA1) [41] with t* = 30 min. Whole brain CB1R availability was computed by creating a composite score from the four lobes and the cerebellum, weighted according to their relative sizes in MNI space, as defined by the AAL atlas.

### Data analysis

We first assessed the association of CB1R availability ([^11^C]OMAR *V*_T_) with sex, age, and BMI in the whole brain and areas postulated to be at the core of PTSD (i.e., amygdala, hippocampus, and frontal cortex) using Bayesian robust regression. Sex, Z-transformed age, and Z-transformed BMI were used as predictors, and minimally informed priors were applied:

$${Intercept} \sim {Normal}(0,1)$$

$${Sex} \sim {Normal}(0,1)$$

$${{Age}}_{Z} \sim {Normal}(0,1)$$

$${{BMI}}_{Z} \sim {Normal}(0,1)$$

To account for outliers, we modeled the outcome variable ([^11^C]OMAR *V*_T_) using a Student’s t distribution [42]:

$${V}_{T} \sim {Student}{\rm{\mbox{'}}}s\,t(\mu ,\nu ,\varepsilon )$$

$$\nu \sim {Inverse\; Gamma}(3,1)$$

$$\varepsilon \sim {Exponential}(1)$$

Next, we tested group effects on CB1R availability in the same brain regions using Bayesian robust regression analysis. Groups were coded as dummy variables, with healthy control (HC) males as the reference. Covariates included sex, Z-transformed age, and Z-transformed BMI. We also added interaction terms for each group with sex, following previous work [16]. Minimally informed priors were used:

$${Intercept} \sim {Normal}\left(0,1\right)$$

$${TC} \sim {Normal}\left(0,1\right)$$

$${HC} \sim {Normal}\left(0,1\right)$$

$${Sex} \sim {Normal}\left(0,1\right)$$

$${{Age}}_{Z} \sim {Normal}\left(0,1\right)$$

$${{BMI}}_{Z} \sim {Normal}\left(0,1\right)$$

$${TC}* {Sex} \sim {Normal}\left(0,1\right)$$

$${HC}* {Sex} \sim {Normal}\left(0,1\right)$$

As with the first analysis, *V*_T_ was modeled using a Student’s t distribution with the same priors:

$${V}_{T} \sim {Student}{\rm{\mbox{'}}}s\,t(\mu ,\nu ,\varepsilon )$$

$$\nu \sim {Inverse\; Gamma}(3,1)$$

$$\varepsilon \sim {Exponential}(1)$$

For the symptom cluster analysis, CAPS scores from 44 participants (19 PTSD; 25 TC) were clustered based on the DSM-5 model and the more robust 8-factor model [30]. Using a similar model to the initial regression model, with Z-transformed age and BMI, and sex, using separate models, we estimated the contribution of each symptom cluster to the whole brain [^11^C]OMAR volume of distribution (*V*_T_).

A robust association was defined as one where the 89% Highest Posterior Density (HPD) interval for the slope did not include 0 [43–45]. All models converged with rHat < 1.01 and effective sample sizes > 1000. The analyses were conducted in Python 3.10.11 using ‘PyMC’ (v4.1.7) [46] and ‘ArviZ’ (v0.17.1) [47]. The No-U-Turn Sampler (NUTS) was employed for Markov chain Monte Carlo (MCMC) inference using default settings: 1000 draws, 1000 tuning steps, an 80% acceptance rate, and no thinning. Additional analyses, including NHPT, were performed with the ‘Pingouin’ package (v0.5.4). All code is available at https://github.com/KoremNSN/CB1rPTSD.

## Results

Demographic and clinical characteristics of the samples are presented in Table 1. No differences were found in [^11^C]OMAR injection parameters, see Supplementary Results for V_T_ values; however, there were differences in age, sex, and body mass index. The HC group was significantly younger and had lower BMI. The TC had a higher male ratio. The PTSD group had higher CAPS scores compared to TC.

### No association between CB1R availability and age, sex, or BMI

A robust regression analysis assessing the association between CB1R availability and age, sex, and BMI, independent of diagnosis/trauma exposure, revealed no evidence of such associations in the amygdala, hippocampus, frontal cortex, or whole brain composite score (See Table 2 for statistics).

**Table 2:** Sex, Age, and BMI Beta Coefficients Contributions to [^11^C]OMAR Volume of Distribution (*V*_T_) Values in Regions of Interest.

Region	Measure	*β* Mean	SD	Lower Bound (5.5%)	Upper Bound (94.5%)
Amygdala	Sex	0.092	0.083	−0.043	0.221
	Age	−0.011	0.037	−0.070	0.046
	BMI	0.025	0.034	−0.029	0.080
Hippocampus	Sex	0.093	0.066	−0.013	0.195
	Age	−0.014	0.033	−0.068	0.039
	BMI	0.033	0.028	−0.012	0.078
Frontal	Sex	0.034	0.067	−0.077	0.137
	Age	−0.005	0.031	−0.052	0.046
	BMI	0.019	0.029	−0.025	0.065
Whole Brain	Sex	0.022	0.063	−0.079	0.120
	Age	−0.007	0.029	−0.052	0.040
	BMI	0.016	0.025	−0.024	0.057

### No association between CB1R availability and group

A robust regression analysis assessing the relationship between CB1R availability and group revealed no evidence of a robust difference in CB1R availability between the groups. However, there was a robust interaction between sex and the TC group in the amygdala, showing increased CB1R availability in females, though this group consisted of only two individuals (see Table 3 for detailed statistics).

**Table 3:** Group Level and Interaction Beta Coefficients Contributions to [^11^C]OMAR volume of distribution (*V*_T_) values in Regions of Interest.

Region	Measure	*β* Mean	SD	Lower Bound (5.5%)	Upper Bound (94.5%)
Amygdala	TC	−0.059	0.104	−0.225	0.107
	PTSD	0.030	0.128	−0.181	0.226
	Sex * TC	**0.388**	**0.229**	**0.038**	**0.759**
	Sex *PTSD	−0.115	0.175	−0.381	0.177
Hippocampus	TC	−0.134	0.091	−0.277	0.009
	PTSD	−0.048	0.104	−0.227	0.100
	Sex * TC	0.254	0.188	−0.021	0.571
	Sex *PTSD	−0.102	0.154	−0.358	0.129
Frontal	TC	−0.090	0.088	−0.236	0.043
	PTSD	−0.002	0.096	−0.163	0.141
	Sex * TC	0.284	0.193	−0.033	0.575
	Sex *PTSD	−0.109	0.141	−0.338	0.111
Whole Brain	TC	−0.073	0.088	−0.214	0.064
	PTSD	0.002	0.099	−0.148	0.169
	Sex * TC	0.263	0.183	−0.032	0.547
	Sex *PTSD	−0.082	0.141	−0.312	0.134

### CB1R availability is associated with symptoms of Anhedonia

Looking at associations between whole brain CB1R availability and symptom clusters, a robust regression analysis showed that only the Anhedonia/emotional numbing cluster was associated with CB1R availability (see Table 4 for detailed statistics).

**Table 4:** Symptom Level Beta Coefficients Contributions to [^11^C]OMAR volume of distribution (*V*_T_) values in the whole brain.

Cluster	*β* Mean	SD	Lower Bound (5.5%)	Upper Bound (94.5%)
Total	0.001	0.002	−0.003	0.005
DSM-5				
B-Intrusion	0.0	0.009	−0.017	0.013
C-Avoidance	0.007	0.015	−0.017	0.03
D-Alteration in mood and cognition	0.004	0.004	−0.003	0.011
E-alteration in arousal	0.003	0.01	−0.013	0.018
8-Cluster				
Internal-Intrusion	−0.004	0.015	−0.029	0.018
External-Intrusion	0.007	0.018	−0.022	0.039
Avoidance	0.007	0.015	−0.018	0.031
Negative Affect	−0.003	0.009	−0.018	0.031
**Anhedonia/Emotional Numbing**	**0.014**	**0.007**	**0.002**	**0.025**
Externalizing Behavior	0.024	0.046	−0.049	0.101
Anxious Arousal	0.017	0.018	−0.012	0.046
Dysphoric Arousal	−0.007	0.016	−0.032	0.018

## Discussion

This study aimed to investigate the associations between CB1R availability and the diagnosis and symptom clusters of PTSD. Unlike Neumeister et al. [16], we did not find differences in CB1R availability in either trauma-exposed individuals or those diagnosed with PTSD, compared to healthy controls. When examining the relationship between PTSD symptom cluster severity and [^11^C]OMAR *V*_T_, we found a robust association with the anhedonia symptom cluster of PTSD, where higher CB1R availability was associated with greater severity of anhedonia/emotional numbing (AN) symptoms. This finding replicates previous results in a larger sample, though with considerable overlap between samples [48]. Further, we found evidence for an interaction effect where women trauma controls showed higher [^11^C]OMAR V_T_ levels compared to healthy men controls. However, this finding requires replication in a larger sample. Overall, these data suggest that, while CB1R availability is not linked to PTSD diagnosis, it is associated with AN symptoms of this disorder.

In general, the ECS is a highly adaptive system capable of dynamically adjusting to physiological demands over a short period of time [29]. Most preclinical studies on the impact of trauma on the EC system measure CB1R expression within a month of trauma exposure, often with continuous stress, to sustain the changes in receptor expression [17, 18]. In humans with PTSD, however, there is usually a period of months to years between the occurrence of the traumatic events and the PTSD diagnosis. In our study, all participants had been diagnosed with PTSD at least one year prior, increasing the likelihood that the ECS had self-regulated and returned to pre-trauma levels over time. The adaptability of ECS has been reported in human studies. For example, among individuals with cannabis dependence, reduced CB1R availability was no longer observed following 28 days of abstinence [12]. Furthermore, the endocannabinoid system’s ability to return to homeostasis might explain why cannabis use does not provide long-term symptom relief in chronic PTSD [49, 50]. While cannabis is often used by trauma-exposed individuals [51], its benefits are typically short-lived, offering brief reductions in symptoms that may also increase AN, particularly regarding pain and anxiety. Nevertheless, the ECS is a complex network that interacts with other physiological systems. Notably, month-long abstinence from alcohol has been associated with persistent downregulation of CB1R availability [29], indicating that factors beyond trauma alone may influence receptor regulation. Another consideration is the timing of trauma exposure, as preliminary research suggests that CB1R expression may differ based on the onset of trauma [24, 52]. Moreover, other characteristics of trauma, such as repetition and chronicity, may play a crucial role, such as low CB1R levels in animal models of chronic stress [21, 22]. Further research is needed to examine links between trauma characteristics, associated disorders, and CB1R availability.

The consistent association between AN and CB1R availability could partly reflect the nature of AN as a persistent defense mechanism [45, 48, 53]. Unlike other PTSD symptom clusters, which may arise more acutely in response to specific triggers, AN could represent a chronic state aimed at suppressing emotional responses to avoid distress [48, 53]. This sustained activation may involve alterations in the ECS, particularly in CB1R availability, to maintain emotional disengagement [54]. Additionally, pre-trauma baseline differences in endocannabinoid signaling could predispose specific individuals to be more vulnerable to developing PTSD and to develop certain symptoms [55] or to rely on emotional numbing as a coping mechanism, possibly making it more consistently linked to CB1R levels over time.

While the sample size and population are comparable between this study and Neumeister et al., [16], several key differences may account for the discrepancy in results. One major factor is trauma type. Neumeister et al.,‘s sample included individuals who had experienced non-combat trauma, while our sample predominantly consisted of veterans. Military-related trauma has been shown to uniquely impact symptom expression [56], cognitive functioning [57], and emotion regulation [58], all of which can influence outcomes and treatment responses [59]. Another notable difference is age; our participants were older than those in Neumeister’s study. Although we did not observe a significant association between age and CB1R availability, prior research suggests that age can weakly influence outcomes and may contribute to variability in results [60]. Moreover, we did not assess plasma cortisol or AEA levels, which were found to differ between PTSD and control groups in Neumeister et al. [16]. While those measures showed predictive value for PTSD diagnosis, a direct link between peripheral levels of endocannabinoids or cortisol and central CB1R availability has not been established.

Several limitations of this study should be noted. The small number of female TC limited our ability to explore sex-by-group interactions in greater depth. While previous studies have shown sex differences using a small number of participants [34], they were not replicated in larger samples [60]. Additionally, initial differences in age and sex across groups may obscure potential interactions with demographic variables. Notably, our PTSD and TC groups were older, and age has been previously associated with reduced CB1R availability. Nevertheless, we did not find a robust effect for age, age-by-group interaction, or an age effect within the healthy control (HC) group.

In conclusion, our results suggest that while CB1R availability is not linked to PTSD diagnosis, it is associated with AN symptoms of this disorder. Our findings align with previous research demonstrating the resilience of the ECS, underscoring the need for more nuanced cannabinoid-based interventions. Future research should aim to replicate these findings with greater female representation in the trauma-exposed control group, as well as a more detailed exploration of the interaction between trauma type and ECS function.

## Supplementary information

Supplementary

## Data Availability

Group-level CB1 V_T_ values used in this study are provided in the Supplementary Materials. Due to institutional and participant privacy restrictions, individual-level demographic, clinical, and MRI data cannot be made publicly available. Additional data supporting the findings of this study may be made available from the corresponding author upon reasonable request and subject to institutional data sharing agreements.
