# Supplementary material for: Cannabinoid 1 receptor availability in posttraumatic stress disorder: A positron emission tomography study
Source: Transl Psychiatry. 2025 Aug 22;15:310. doi: 10.1038/s41398-025-03519-9 (PMC12373994; doi:10.1038/s41398-025-03519-9)
Supplement: Supplementary file 1 — Supplementary [file 41398_2025_3519_MOESM1_ESM.docx]

# Supplementary methods

## Inclusion and exclusion criteria

**Inclusion criteria across all study populations:**

1) Gender: Both males and females.

2) Age: between the ages of 18 and 70 years, inclusive.

3) Have clinical laboratory test results within normal reference range for the population or investigator site, or results with acceptable deviations that are judged to be not clinically significant by the investigator.

4) Have arterial access sufficient to allow blood sampling as per the protocol.

5) Are reliable and willing to make themselves available for the duration of the study, and are willing to follow study procedures.

6) Have given written informed consent

**Inclusion criteria for the PTSD population:**

1) Meets criteria for PTSD diagnosis as defined by the DSM

**Exclusion criteria across all study populations**

1) Current non-affective (MDD) neuro-psychiatric illness, psychotic disorders, moderate (and above) substance abuse, or severe systemic disease based on history and physical exam. Nicotine dependence will be permitted.

2) Laboratory tests with clinically significant abnormalities or a positive urine toxicology screen.

3) Prior participation in other research protocols in the last year, such that radiation exposure would exceed the annual limits.

4) Presence of ferromagnetic metal in the body or a heart pacemaker.

5) Women with a positive pregnancy test or women who are lactating.

6) Have a history of head trauma with prolonged loss of consciousness (>30 minutes), or any neurological condition, including stroke or seizure disorder (excluding a single childhood febrile seizure)

7) Current treatment with psychotropic medications, other than a stable dose for 2 months of antidepressants, except for allowances made at the discretion of the PI.

8) Have used any prescription medication except for oral contraceptives or those approved by the investigator.

9) Have implanted or embedded metal objects or fragments in the head or body that would present a risk during the MRI scanning procedure, or have worked with ferrous metals either as a vocation or hobby (for example, as a sheet metal worker, welder, or machinist) in such a way that might have led to unknown, indwelling metal fragments that could cause injury if they moved in response to placement in the magnetic field

10) Have had exposure to ionizing radiation that, in combination with the study tracer, would result in a cumulative exposure that exceeds recommended exposure limits.

11) Are claustrophobic.

12) Have a history of a bleeding disorder, or use of medication to thin blood.

13) Failure of the Allen’s test upon screening.

## The 8-Cluster scoring table

Loading tables of PCL-5 items based on Duek et al., 2022.

Items 1-3 - Intrusion symptoms internal

Items 4-5 - Intrusion symptoms external

Items 6-7- Avoidance

Items 8-11 - Negative affect

Items 12-14 - Anhedonia / emotional numbing

Items 15-16 - Externalizing behaviors

Items 17-18 - Anxious arousal

Items 19-20 - Dysphoric arousal

Duek, O., Spiller, T. R., Rubenstein, A., Pietrzak, R. H., & Harpaz-Rotem, I. (2022). Exploration of a novel model of intrusive symptoms in posttraumatic stress disorder among US veterans. *JAMA Network Open*, *5*(3), e223555-e223555.

# Supplementary results

To directly replicate the analyses from Neumeister et al., we conducted several additional analyses. A bivariate correlation analysis revealed no significant correlations between composite [^11^C]OMAR V_T_ values and age (r = −0.07, P = 0.58), sex (r = −0.08, P = 0.53; Pearson's correlation was used despite categorical variables), or BMI (r = 0.05, P = 0.68). An ANCOVA examining mean composite [11C]OMAR VT values across HC, TC, and PTSD groups showed no significant main effects of group, sex, or age, and no significant two- or three-way interactions (all p > 0.16). Similarly, no effects were observed in the amygdala, hippocampus, or frontal cortex, brain regions that comprise the neural circuit implicated in PTSD.


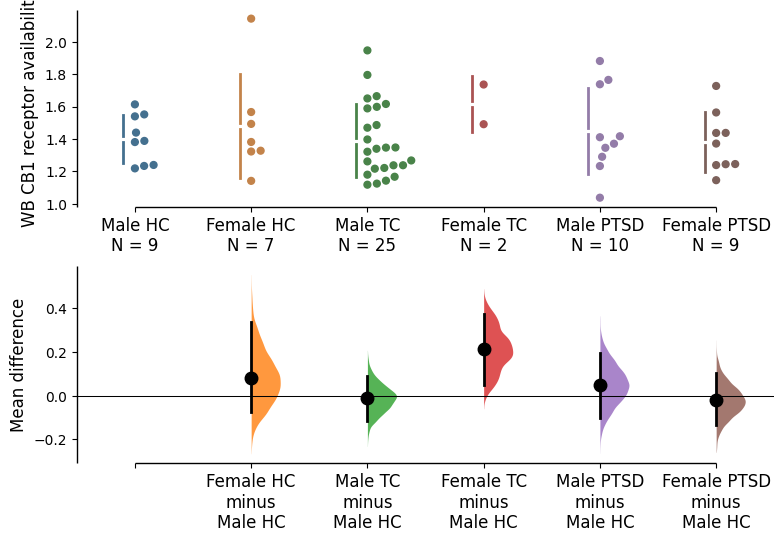


Cannabinoid 1 Receptor VT values per region of interest

| PatientID# | amygdala | caudate | cerebellum | cingulum_ant | cingulum_post | frontal | hippocampus | insula | occipital | parietal | putamen | temporal | thalamus | Group |
| --- | --- | --- | --- | --- | --- | --- | --- | --- | --- | --- | --- | --- | --- | --- |
| 1 | 1.23 | 0.99 | 1.03 | 1.31 | 0.97 | 1.17 | 1.19 | 1.24 | 1.06 | 1.04 | 1.36 | 1.14 | 0.9 | HC |
| 2 | 1.26 | 0.93 | 1.06 | 1.22 | 0.83 | 1.19 | 1.13 | 1.15 | 1.01 | 1.2 | 1.25 | 1.07 | 0.9 | Yes |
| 3 | 1.61 | 1.22 | 1.47 | 1.66 | 1.31 | 1.47 | 1.42 | 1.62 | 1.33 | 1.31 | 1.68 | 1.49 | 1.15 | Yes |
| 4 | 1.3 | 1.06 | 1.02 | 1.38 | 1 | 1.32 | 1.18 | 1.32 | 1.23 | 1.24 | 1.49 | 1.28 | 0.96 | HC |
| 5 | 1.05 | 0.86 | 1.05 | 1.08 | 0.85 | 1.01 | 0.9 | 1.07 | 0.97 | 1.05 | 1.12 | 1.05 | 0.7 | No |
| 6 | 1.15 | 0.9 | 1.06 | 1.16 | 0.87 | 1.14 | 1.05 | 1.14 | 1.11 | 1.11 | 1.23 | 1.16 | 0.84 | HC |
| 7 | 1.11 | 0.75 | 1.09 | 1.25 | 0.91 | 1.11 | 1.01 | 1.21 | 1.1 | 1.1 | 1.27 | 1.14 | 0.92 | No |
| 8 | 1.88 | 1.48 | 1.49 | 1.76 | 1.33 | 1.65 | 1.49 | 1.7 | 1.52 | 1.5 | 1.84 | 1.6 | 1.28 | No |
| 9 | 1.08 | 0.94 | 1.13 | 1.3 | 0.93 | 1.24 | 1.12 | 1.26 | 1.07 | 1.12 | 1.34 | 1.19 | 0.94 | Yes |
| 10 | 1.24 | 1.04 | 1.11 | 1.34 | 0.95 | 1.18 | 1.16 | 1.31 | 1.13 | 1.04 | 1.39 | 1.21 | 1.03 | No |
| 11 | 1.44 | 1.31 | 1.3 | 1.67 | 1.03 | 1.53 | 1.39 | 1.6 | 1.32 | 1.3 | 1.77 | 1.48 | 1.27 | HC |
| 12 | 1.75 | 1.33 | 1.66 | 1.97 | 1.3 | 1.79 | 1.55 | 1.87 | 1.79 | 1.75 | 1.95 | 1.78 | 1.3 | No |
| 13 | 1.38 | 1.15 | 1.27 | 1.43 | 1.12 | 1.3 | 1.34 | 1.4 | 1.2 | 1.2 | 1.53 | 1.34 | 1.1 | No |
| 14 | 2 | 1.5 | 1.58 | 2 | 1.59 | 1.74 | 1.67 | 1.95 | 1.66 | 1.71 | 2.02 | 1.75 | 1.45 | Yes |
| 15 | 1.46 | 1.13 | 1.31 | 1.59 | 1.24 | 1.55 | 1.3 | 1.62 | 1.33 | 1.42 | 1.59 | 1.55 | 1.2 | No |
| 16 | 1.21 | 0.97 | 1 | 1.23 | 0.93 | 1.1 | 1.05 | 1.18 | 0.98 | 0.98 | 1.26 | 1.05 | 0.88 | HC |
| 17 | 1.9 | 1.29 | 1.52 | 1.83 | 1.28 | 1.68 | 1.49 | 1.78 | 1.54 | 1.63 | 1.84 | 1.67 | 1.22 | No |
| 18 | 1.02 | 0.74 | 1.02 | 1.15 | 0.7 | 1.07 | 0.91 | 1.11 | 0.94 | 0.96 | 1.11 | 1.04 | 0.8 | No |
| 19 | 1.55 | 1.45 | 1.44 | 1.65 | 1.29 | 1.45 | 1.5 | 1.71 | 1.38 | 1.45 | 1.81 | 1.59 | 1.18 | HC |
| 20 | 1.12 | 0.84 | 1.07 | 1.27 | 0.97 | 1.17 | 1.1 | 1.31 | 1.1 | 1.04 | 1.31 | 1.17 | 0.9 | No |
| 21 | 1.64 | 1.13 | 1.26 | 1.65 | 1.32 | 1.46 | 1.49 | 1.65 | 1.29 | 1.38 | 1.68 | 1.51 | 1.1 | HC |
| 22 | 1.28 | 1.03 | 1.14 | 1.38 | 1.09 | 1.29 | 1.02 | 1.35 | 1.19 | 1.17 | 1.44 | 1.33 | 1 | Yes |
| 23 | 1.48 | 1.23 | 1.26 | 1.46 | 1.13 | 1.32 | 1.38 | 1.47 | 1.2 | 1.31 | 1.58 | 1.38 | 1.14 | Yes |
| 24 | 1.2 | 0.87 | 0.96 | 1.16 | 0.89 | 1.05 | 0.93 | 1.13 | 1.06 | 0.98 | 1.27 | 1.09 | 0.81 | No |
| 25 | 1.36 | 1.1 | 1.17 | 1.43 | 0.99 | 1.29 | 1.26 | 1.39 | 1.18 | 1.29 | 1.42 | 1.26 | 1.01 | HC |
| 26 | 1.34 | 1 | 1.38 | 1.38 | 0.94 | 1.3 | 1.17 | 1.36 | 1.26 | 1.31 | 1.28 | 1.34 | 0.97 | HC |
| 27 | 1.27 | 0.89 | 1.04 | 1.29 | 0.85 | 1.15 | 1.06 | 1.18 | 1.1 | 1.19 | 1.21 | 1.11 | 0.86 | Yes |
| 28 | 1.08 | 0.82 | 1.15 | 1.22 | 0.86 | 1.21 | 0.96 | 1.18 | 1.1 | 1.11 | 1.24 | 1.13 | 0.91 | No |
| 29 | 0.95 | 0.79 | 1.18 | 1.2 | 0.81 | 1.15 | 1.09 | 1.16 | 1.01 | 1.13 | 1.21 | 1.16 | 0.83 | No |
| 30 | 1.2 | 0.9 | 1.04 | 1.26 | 0.99 | 1.11 | 1.04 | 1.26 | 1.13 | 1.04 | 1.33 | 1.17 | 0.96 | No |
| 31 | 1.92 | 1.79 | 1.99 | 2.06 | 1.97 | 1.96 | 1.89 | 1.98 | 1.8 | 2.02 | 2.2 | 1.97 | 1.48 | HC |
| 32 | 1.59 | 1.26 | 1.42 | 1.63 | 1.33 | 1.56 | 1.51 | 1.63 | 1.43 | 1.43 | 1.62 | 1.54 | 1.18 | No |
| 33 | 1.33 | 0.8 | 1.23 | 1.4 | 1.04 | 1.37 | 1.19 | 1.29 | 1.2 | 1.23 | 1.39 | 1.29 | 0.88 | Yes |
| 34 | 1.26 | 0.81 | 1.28 | 1.15 | 1.07 | 1.29 | 1 | 1.34 | 1.33 | 1.57 | 1.43 | 1.34 | 0.86 | No |
| 35 | 1.42 | 0.99 | 1.21 | 1.41 | 0.98 | 1.29 | 1.22 | 1.4 | 1.14 | 1.22 | 1.38 | 1.29 | 0.94 | Yes |
| 36 | 1.17 | 0.9 | 1.17 | 1.31 | 0.87 | 1.27 | 1.13 | 1.23 | 1.15 | 1.2 | 1.24 | 1.22 | 0.94 | No |
| 37 | 1.33 | 0.8 | 1.17 | 1.36 | 1.02 | 1.24 | 1.13 | 1.28 | 1.27 | 1.1 | 1.42 | 1.3 | 0.96 | Yes |
| 38 | 1.38 | 0.93 | 0.97 | 1.43 | 0.97 | 1.3 | 1.02 | 1.38 | 1.23 | 1.09 | 1.32 | 1.31 | 0.88 | No |
| 39 | 1.58 | 1.43 | 1.52 | 1.71 | 1.42 | 1.63 | 1.57 | 1.62 | 1.45 | 1.56 | 1.82 | 1.59 | 1.28 | Yes |
| 40 | 0.74 | 0.71 | 0.86 | 0.98 | 0.74 | 0.98 | 0.71 | 0.9 | 0.91 | 0.98 | 0.96 | 0.9 | 0.74 | Yes |
| 41 | 1.63 | 1.33 | 1.39 | 1.56 | 1.12 | 1.59 | 1.43 | 1.68 | 1.56 | 1.61 | 1.71 | 1.64 | 1.16 | Yes |
| 42 | 1.57 | 1.12 | 1.28 | 1.7 | 1.13 | 1.55 | 1.4 | 1.58 | 1.41 | 1.57 | 1.69 | 1.58 | 1.08 | No |
| 43 | 1.26 | 1.11 | 1.13 | 1.25 | 1.2 | 1.23 | 1.25 | 1.29 | 1.19 | 1.21 | 1.45 | 1.2 | 0.95 | HC |
| 44 | 1.26 | 1.04 | 1.32 | 1.25 | 1.15 | 1.36 | 1.24 | 1.35 | 1.21 | 1.42 | 1.37 | 1.33 | 1.03 | No |
| 45 | 1.43 | 1.09 | 1.2 | 1.41 | 1.08 | 1.28 | 1.21 | 1.33 | 1.13 | 1.16 | 1.38 | 1.27 | 0.95 | No |
| 46 | 1.35 | 0.97 | 0.99 | 1.26 | 0.88 | 1.13 | 1.13 | 1.25 | 1 | 0.99 | 1.29 | 1.08 | 1.01 | No |
| 47 | 1.11 | 0.82 | 1 | 1.25 | 0.92 | 1.2 | 1.08 | 1.22 | 1.08 | 1.06 | 1.28 | 1.17 | 0.86 | Yes |
| 48 | 1.18 | 0.91 | 1.17 | 1.31 | 1.06 | 1.22 | 1.24 | 1.36 | 1.16 | 1.17 | 1.36 | 1.24 | 0.94 | No |
| 49 | 1.36 | 1.29 | 1.3 | 1.49 | 1.16 | 1.44 | 1.39 | 1.52 | 1.36 | 1.4 | 1.53 | 1.41 | 1.08 | HC |
| 50 | 1.61 | 1.27 | 1.28 | 1.54 | 1.17 | 1.4 | 1.38 | 1.57 | 1.27 | 1.33 | 1.63 | 1.41 | 1.16 | HC |
| 51 | 1.24 | 0.8 | 1.12 | 1.22 | 0.88 | 1.03 | 0.94 | 1.11 | 1.08 | 1.08 | 1.13 | 1.12 | 0.84 | No |
| 52 | 1.57 | 1.22 | 1.34 | 1.59 | 1.25 | 1.4 | 1.34 | 1.58 | 1.24 | 1.23 | 1.58 | 1.48 | 1.08 | No |
| 53 | 1.35 | 1.05 | 1.17 | 1.35 | 1.09 | 1.27 | 1.25 | 1.35 | 1.11 | 1.11 | 1.5 | 1.25 | 1.09 | HC |
| 54 | 1.49 | 1.13 | 1.18 | 1.45 | 1.22 | 1.36 | 1.41 | 1.42 | 1.24 | 1.3 | 1.5 | 1.32 | 1.09 | Yes |
| 55 | 1.36 | 1.14 | 1.26 | 1.41 | 1.16 | 1.28 | 1.19 | 1.42 | 1.21 | 1.22 | 1.39 | 1.31 | 1.02 | HC |
| 56 | 1.07 | 0.83 | 1.06 | 1.2 | 0.94 | 1.16 | 1.03 | 1.2 | 1.06 | 1.08 | 1.22 | 1.16 | 0.8 | HC |
| 57 | 1.36 | 1.34 | 1.41 | 1.5 | 1.21 | 1.48 | 1.38 | 1.63 | 1.32 | 1.41 | 1.63 | 1.52 | 1.15 | No |
| 58 | 1.6 | 1.27 | 1.49 | 1.8 | 1.26 | 1.62 | 1.46 | 1.67 | 1.61 | 1.71 | 1.76 | 1.54 | 1.15 | Yes |
| 59 | 1.66 | 1.11 | 1.19 | 1.61 | 1.24 | 1.51 | 1.35 | 1.61 | 1.34 | 1.59 | 1.6 | 1.44 | 1.02 | No |
| 60 | 1.6 | 1 | 1.36 | 1.48 | 1.15 | 1.26 | 1.3 | 1.47 | 1.28 | 1.23 | 1.52 | 1.33 | 1.1 | Yes |
| 61 | 0.91 | 0.79 | 1.03 | 0.96 | 0.83 | 1 | 0.9 | 0.96 | 1.13 | 1.06 | 1.02 | 1.05 | 0.76 | Yes |
| 62 | 1.06 | 0.95 | 1 | 1.17 | 0.86 | 1.13 | 1 | 1.16 | 1.11 | 1.17 | 1.28 | 1.12 | 0.79 | Yes |
